# Comparison of three doses of amikacin on alternate days with a daily dose of meropenem during the same period for the treatment of urinary tract infection with *E. coli*: a double-blind clinical trial

**DOI:** 10.1186/s13104-023-06654-y

**Published:** 2024-01-25

**Authors:** Behzad Mohsenpour, Amjad Ahmadi, Hero Azizzadeh, Ebrahim Ghaderi, Katayon Hajibagheri, Shahla Afrasiabian, Gohar Lotfi, Zhila Farzinpoor

**Affiliations:** 1 Zoonoses Research Center, Research Institute for Health Development, Kurdistan University of Medical Sciences, Sanandaj, Iran; 2 Department of Infectious Diseases, Faculty of Medicine, Kurdistan University of Medical Sciences, Sanandaj, Iran; 3 Department of Microbiology, School of Medicine, Hamadan University of Medical Sciences, Hamadan, Iran; 4 Student Research Committee, Kurdistan University of Medical Sciences, Sanandaj, Iran; 5 Social Determinants of Health Research Center, Kurdistan University of Medical Sciences, Sanandaj, Iran

**Keywords:** Amikacin, Meropenem, *E. Coli*, ESBL, UTIs

## Abstract

**Objectives:**

Urinary tract infections (UTIs) are very common infections in humans, and *Escherichia coli* (*E. coli*) is the commonest pathogen leading to UTIs. The generation of beta-lactamase enzymes in this bacterium results in its resistance against many antibiotics. This study compares three doses of amikacin on alternate days with a daily dose of meropenem in the same period for the treatment of UTIs with *E. coli* in a double-blind clinical trial.

**Methods:**

The current double-blind clinical trial compares three doses of amikacin on alternate days with a daily dose of meropenem in the same period for the treatment of UTIs with *E. coli.* The patients were assigned to two groups: Intervention (receiving a single dose of amikacin once a day at 48-h intervals for a week, three doses) and control (receiving meropenem for 1/TDS for a week).

**Results:**

The *E. coli* infection frequency was 61 (21 cases of non-ESBL and 40 cases of ESBL-positive infections) and the frequency of the other infections was 52 (46%). In the patients with ESBL *E. coli* infection, ciprofloxacin (21; 70%) showed the highest antibiotic resistance, and nitrofurantoin (33; 91.7%) showed the highest sensitivity. The baseline variables between the control and intervention groups indicated no significant difference (p > 0.05). The frequency of signs and symptoms showed no significant difference between the amikacin and meropenem groups in the first 24 h and the first week. In the second week of follow-up, no clinical signs or symptoms were observed in the two groups.

**Conclusion:**

The results of this study showed that treatment with amikacin, 1 g q48h, for one week (three doses) has the same result as meropenem, 1 g q8h, for one week (21 doses). The results are the same for the treatment of UTIs with ESBL positive and ESBL negative. Amikacin can be used once every 48 h to treat UTIs, is less expensive and can be administered on an outpatient basis.

**Trial registration:**

This study was registered in the Iranian Registry of Clinical Trials (IRCT) with ID number: IRCT20170417033483N2 on the date 2018-02-13.

## Introduction

Urinary tract infections (UTIs) are very common infections in humans, and *Escherichia coli* (*E. coli*) is the main cause of UTIs [1, 2]. *E. coli* as a Gram-negative *bacillus* belongs to the *Enterobacteriaceae* family. Treating infections caused by *E. coli* is challenging due to antibiotic-resistant strains. The generation of extended spectrum beta-lactamas (ESBL) in *E. coli* causes its resistance against several antibiotics [3, 4]. Beta lactamases can be generated by Gram-negative bacteria and are present in the *Enterobacteriaceae* family. ESBL-producing bacteria are resistant to cephalosporins, penicillin, tazobactam/piperacillin and other antibiotics such as co-trimoxazole, fluoroquinolones, and tetracycline. Also, the ESBL-coding plasmid easy transfer is an important threat to hospitalized patients [5, 6]. Recently, numerous global studies regarding the antibiotic resistance of *E. coli* indicated the resistance of many medications and ESBL [7–9]. The multidrug resistance level was 7.1% and 10.9% in the US, and Iran, respectively [10, 11]. Researchers are investigating alternative drugs, like aminoglycosides, fosfomycin, carbapenems and piperacillin/tazobactam. Carbapenems are the treatment of choice for such organisms, however, *Enterobacteriaceae* is resistant to carbapenems [12]. The accepted treatment is using carbapenems and patients should be hospitalized to receive this treatment. Hospitalization can have consequences and lead to loss of working days and thus increase the costs incurred to the patient as well as the risk of hospital-acquired infections. Using aminoglycosides has decreased recently [1], mainly because of their side effects, which are greater than other antibiotics [13]. Nevertheless, patients treated with aminoglycosides experience fewer side effects than those receiving meropenem, as the former does not need hospitalization and has lower nosocomial infection rates and no complications of IV line such as thrombophlebitis [1, 13, 14]. The prevalence of microbial resistance against aminoglycosides has been low recently. Using aminoglycosides is as effective as beta lactams or quinolones in obtaining clinical improvement for UTIs [1, 15–17]. Thus it is essential to find an effective alternative method. This study compares three doses of amikacin on alternate days with a daily dose of meropenem in the same period for the treatment of UTIs with *E. coli* in a double-blind clinical trial.

## Materials and methods

The current double-blind clinical trial was registered at the Iranian Registry of Clinical Trials (IRCT20170417033483N2) date of registration (2018-02-13). Our study statistical community was being a patient with upper or lower UTI and the consequent symptoms who was visited in Tohid Hospital in Sanandaj. Also, the inclusion criteria were: Burning sensation while urinating, frequent urination, pain in the right upper quadrant, fever, and *E. coli* infection. The exclusion criteria were recent use of antibiotics, septic shock, using immunosuppressive drugs, and/or a glomerular filtration rate (GFR) of < 60 mL/min and creatinine level > 3. The patients’ vital signs referring to the hospital were assessed and after a visit from a doctor, urine culture and analysis and a smear test was performed. All diagnostic tests were performed by the microbiology department of the medical diagnosis laboratory of Tohid Hospital. In the case of the urine analysis results indicating the chance of UTI, the patient was included in the research until obtaining a positive culture result based on gram staining, chemical reactions, differential and selective media: Blood Agar, Eosin methylene blue (EMB), Sulfide indol motility (SIM), Triple sugar iron agar (TSI) and Simmon citrate agar (Ibresco Made in Italy (. Cases with a negative culture or multiple bacteria were excluded. Informed consent was obtained before the experiments. The patients were assigned to the intervention and control groups by simple randomization for each type of *E. coli* ESBL positive or non-ESBL (The identification of ESBL was based on the Clinical and Laboratory Standards Institute (CLSI) guidelines and antibiotic disks of Rosco company). Flowchart 1 shows the number of patients in each group. The research was double-blind, as the physician, the patients, and the laboratory personnel were blinded to the group allocation. Randomization and preparation of the medicines were done by a trained nurse.

The intervention group was treated with amikacin 15 mg/kg every 48 h (maximum 1 g) for seven days, followed by ofloxacin 300 mg twice a day for another seven days after the primary injectable treatment (up to day 14 or end of treatment). Clinical signs were noted, and urine culture and analysis were done. The control group was treated with meropenem at 1 g three times a day for a week, followed by ofloxacin 300 mg two times a day for seven days after the primary injectable treatment. Both study groups received the same frequency of injection (drugs or normal saline as a placebo). The drugs were prepared and injected by a microinfusion set.

### Data analysis

Data were analyzed by SPSS 18; quantitative data were reported as mean and SD, whereas, qualitative data as percentage and frequency. An independent t-test compared two quantitative variables. Fisher’s exact test compared the treatment effectiveness between the two groups.

**Flowchart 1:**
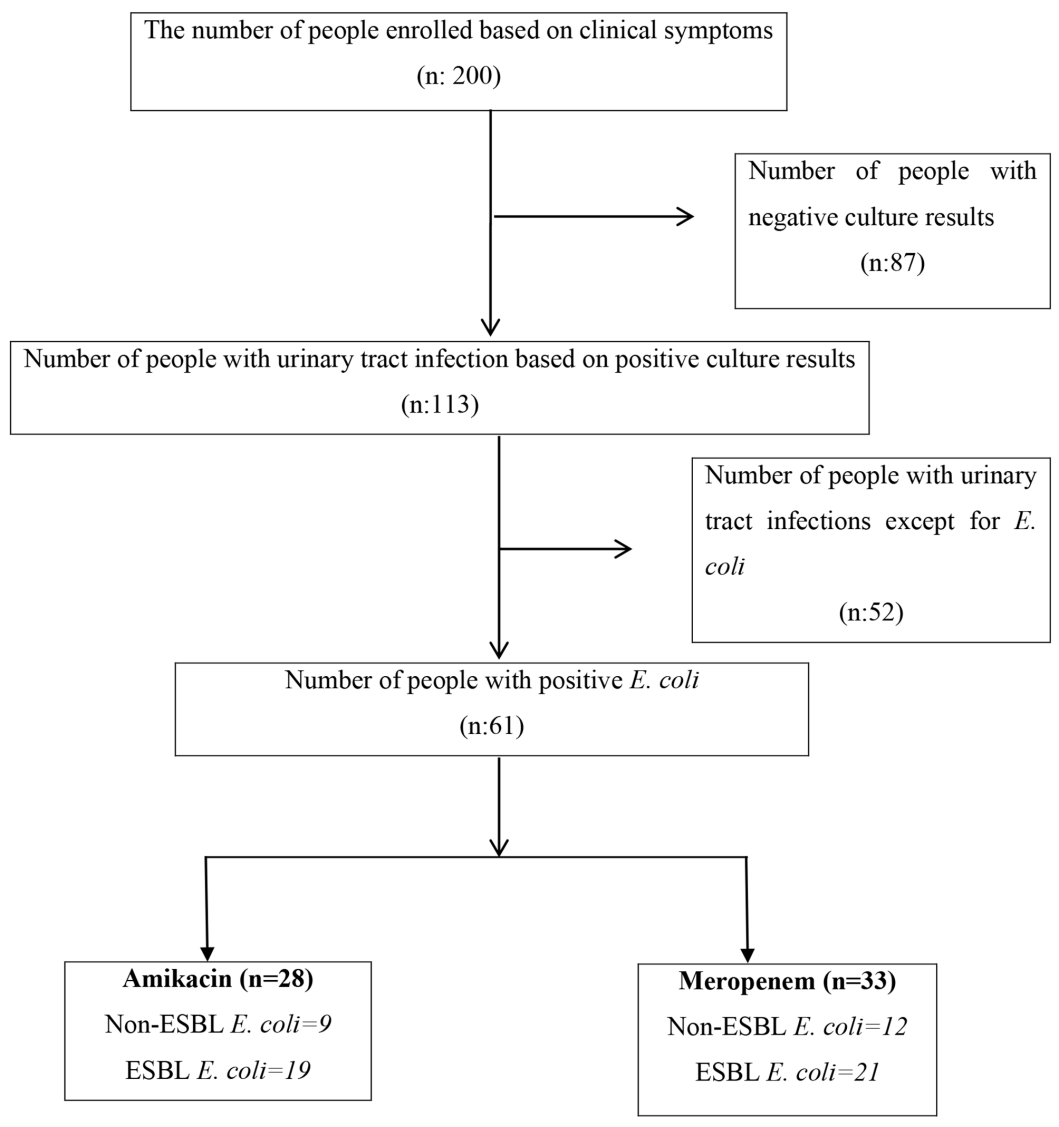
Inclusion of people in the study

## Results

The mean age was 46.64 ± 3.89 years in the intervention group and 46.03 ± 2.38 years in the control group. The frequency of *E. coli* infection was 61 (21 cases non-ESBL and 40 cases ESBL positive), and 52 (46%) for other infections. Five of the men (38.5%) and 35 of the women (72.9%) were ESBL *E. coli* (p = 0.02).

In the ESBL *E. coli* infection patients, the highest antibiotic resistance and sensitivity were reported for ciprofloxacin (21; 70%) and nitrofurantoin (33; 91.7%), respectively. In cases that had non-ESBL *E. coli* infection, the highest antibiotic resistance and sensitivity were related to ciprofloxacin (9; 60%) and nitrofurantoin and gentamicin (13; 86.7%), respectively. Tables 1, 2 and 3 summarize the results. The baseline variables did not show significant differences between the groups (p > 0.05). The frequency of signs and symptoms showed no significant difference between the amikacin and meropenem groups in the first 24 h and the first week (Tables 4 and 5). In the second week of follow-up, no clinical signs or symptoms were observed in the two groups. Also, to assess the ototoxicity and nephrotoxicity before and after receiving amikacin, the patients were followed up regarding clinical signs and symptoms; for example, balance disorder, hearing loss, and vertigo, during and after the treatment. No nephrotoxicity or ototoxicity was observed in any of the patients.

**Table 1:** Frequency of the measured variables before the treatment

Variable	Groups	Value	*P*-value
**Age**	Amikacin	20.5 ± 46.6	0.89✝
	Meropenem	13.6 ± 46.6	
**Female**	Amikacin	21 (75%)	0.51
	Meropenem	27 (81.7%)	
**History of UTI**	Amikacin	14 (50%)	0.55
	Meropenem	19 (57.6%)	
**Dysuria**	Amikacin	25 (89.3%)	1 *
	Meropenem	29 (87.9%)	
**Frequent urination**	Amikacin	25 (89.3%)	1 *
	Meropenem	29 (87.9%)	
**Abdominal pain**	Amikacin	6 (21.4%)	0.08
	Meropenem	14 (42.4%)	
**Flank pain**	Amikacin	12 (42.9%)	0.06
	Meropenem	22 (66.7%)	
**Suprapubic pain**	Amikacin	19 (67.9%)	0.4
	Meropenem	19 (57.6%)	
**Costovertebral angle tenderness (CVAT)**	Amikacin	13 (46.4%)	0.11
	Meropenem	22 (66.7%)	

**Table 2:** Antibiotic resistance in the ESBL *E. coli* group

Antibiotics	Sensitive	Intermediate	Resistant
Ampicillin	1 (2.5%)	2 (5%)	12 (30%)
Gentamicin	18 (45%)	2 (5%)	9 (22.5%)
Piperacillin/Tazobactam	4 (10%)	9 (22.5%)	16 (40%)
Cefepime	5(12.5%)	1 (2.5%)	15 (37.5%)
Cefotaxime	5 (12.5%)	3 (7.5%)	15 (37.5%)
Ceftazidime	7 (17.5%)	3 (7.5%)	12 (30%)
Ciprofloxacin	6 (15%)	3 (7.5%)	2 (5%)
Meropenem	19 (47.5%)	3 (7.5%)	1(2.5%)
Trimethoprim/sulfamethoxazole	4 (10%)	3 (7.5%)	2 (5%)
Nitrofurantoin	33 (82.5%)	3 (7.5%)	0 (0%)
Clavulanic acid + Ceftazidime	3 (7.5%)	0 (0%)	1(2.5%)
Clavulanic acid + Cefotaxime	3 (7.5%)	0 (0%)	1 (2.5%)
Amikacin	10 (25%)	1 (2.5%)	0 (0%)
Oxacillin	0 (0%)	0 (0%)	0 (0%)
Norfloxacin	1 (2.5%)	1 (2.5%)	4 (10%)
Clindamycin	1 (2.5%)	0 (0%)	2 (5%)

**Table 3:** Antibiotic resistance in non-ESBL *E. coli*

Antibiotics	Sensitive	Intermediate	Resistant
Ampicillin	2 (9.5%)	0 (0%)	5 (23.8%)
Gentamicin	13 (61.9%)	1 (4.8%)	3 (14.3%)
Piperacillin/Tazobactam	10 (47.6%)	2 (9.6%)	5 (23.8%)
Cefepime	4 (19%)	0 (0%)	5 (23.8%)
Cefotaxime	3 (14.3%)	0 (0%)	7 (33.3%)
Ceftazidime	2 (9.5%)	1 (4.8%)	6 (28.6%)
Ciprofloxacin	6 (28.6%)	0 (0%)	9 (42.9%)
Meropenem	12 (57.1%)	0 (0%)	2 (9.5%)
Trimethoprim/sulfamethoxazole	5 (23.8%)	0 (0%)	8 (38.1%)
Nitrofurantoin	13 (61.9%)	0 (0%)	2 (9.5%)
Clavulanic acid + Ceftazidime	0 (0%)	0 (0%)	0 (0%)
Clavulanic acid + Cefotaxime	1 (4.8%)	0 (0%)	0 (0%)
Amikacin	6 (28.6%)	0 (0%)	0 (0%)
Oxacillin	3 (14.3%)	0 (0%)	0 (0%)
Norfloxacin	2 (9.5%)	0 (0%)	2 (9.5%)
Clindamycin	1 (4.8%)	0 (0%)	0 (0%)

**Table 4:** Frequency distribution of clinical signs and symptoms following 48 h of treatment

Variable	Groups	Value	*P*-value
**Fever**	Amikacin	0	0.24 *
	Meropenem	3 (9.1%)	
**Dysuria**	Amikacin	4 (14.3%)	1 *
	Meropenem	5 (15.2%)	
**Frequent urination**	Amikacin	3 (10.7%)	0.71 *
	Meropenem	5 (15.2%)	
**Abdominal pain**	Amikacin	1 (3.7%)	0.2 *
	Meropenem	5 (15.2%)	
**Flank pain**	Amikacin	3 (10.7%)	0.2 *
	Meropenem	8 (24.2%)	
**Suprapubic pain**	Amikacin	6 (21.4%)	0.49*
	Meropenem	4 (12.1%)	
**Costovertebral angle tenderness (CVAT)**	Amikacin	0	-
	Meropenem	0	

**Table 5:** Frequency distribution of the measured variables following one week of treatment

Variable	Groups	Value	*P*-value
**Fever**	Amikacin	0	-
	Meropenem	0	
**Dysuria**	Amikacin	2 (7.1%)	0.58 *
	Meropenem	1 (3%)	
**Frequent urination**	Amikacin	2 (7.1%)	0.2 *
	Meropenem	0	
**Abdominal pain**	Amikacin	1 (3.6%)	1 *
	Meropenem	1 (3%)	
**Flank pain**	Amikacin	2 (7.1%)	0.58 *
	Meropenem	1 (3%)	
**Suprapubic pain**	Amikacin	2 (7.1%)	0.58 *
	Meropenem	1 (3%)	
**Costovertebral angle tenderness (CVAT)**	Amikacin	0	-
	Meropenem	0	

## Discussion

A treatment regimen with monotherapy and a single daily dose of amikacin with two-day intervals significantly reduced clinical signs and symptoms of UTIs within 48 h, and one week after the start of treatment. The symptoms and clinical signs of UTIs were resolved after two weeks of treatment in both groups, and both groups showed similar findings regarding reduced *E. coli* UTIs. Although this decrease was more considerable in the control group after a week, both groups showed no *E. coli* infections after treatment for two weeks. Patients treated with aminoglycosides had fewer side effects in comparison with cases receiving beta-lactams. The standard treatment is using carbapenems and patients should be hospitalized, which causes consequences such as increased costs, lost working days, and increased risk of hospital-acquired infections [1, 15–18]. It appears that treatment with amikacin, 1 g q48h, can be a suitable alternative for common treatments of *E. coli* UTIs. In a study conducted by Soltani et al. in 2012 to determine the pattern of antibiotic sensitivity against gram-negative bacteria, the most effective antibiotics against these bacteria were imipenem followed by ciprofloxacin [19]. Cho et al. conducted a clinical trial between 2011 and 2012 in South Korea and examined nine episodes of UTIs due to ESBL *E. coli* in eight women receiving outpatient intravenous treatment with amikacin. The average length of treatment was ten days. The findings indicated laboratory and clinical improvements after the treatment with amikacin in all the episodes and one case of relapse and one untreated case were reported [1]. Their results are consistent with our observations. SH Wie et al. in South Korea showed that gentamicin can be an effective antibiotic for initial empiric treatment of acute pyelonephritis(APN), especially in patients who do not require urological procedures. They also showed that the use of gentamicin may prevent the use of fluoroquinolones or broad-spectrum cephalosporins in the treatment of complicated non-obstructive APN [20]. In a retrospective cohort study by Anderson et al. conducted in 2022, the efficacy of non-carbapenem beta-lactams (NCBL) was compared to carbapenems for UTIs of broad-spectrum beta-lactamase-producing *Enterobacteriaceae*. Patients treated with NCBLs had a similar length of hospital stay, shorter durations of antibiotic therapy, and higher rates of culture clearance than cases receiving carbapenems, which suggests that ESBL UTIs treatment should not be only based on phenotypic resistance [21]. In the clinical trial study conducted by Derkonja et al. in 2021, which examined the effect of 7-day ciprofloxacin or trimethoprim/sulfamethoxazole treatment in patients with febrile symptoms and UTI, using a 7-day course of ciprofloxacin or sulfamethoxazole/trimethoprim was found to be suitable as an alternative for a 14-day course to treat febrile men with UTIs [22]. In another clinical trial study conducted in 2021 by Dorado et al. titled “Effectiveness of Fosfomycin for the Treatment of Multidrug-Resistant Escherichia coli Bacteremic Urinary Tract Infections”, fosfomycin showed no noninferiority in the treatment of bacteremic UTIs due to MDR E coli. Nevertheless, their data showed that fosfomycin can be regarded for those with these infections [23]. Although sensitivity to gentamicin was 83.3% in the cited study, which is higher than that in the current study, both studies reported a high sensitivity to gentamicin. Han et al. in South Korea examined 211 children < 14 years of age diagnosed with UTIs caused by *E. coli* and *Klebsiella pneumoniae* who were referred to clinics outside or inside hospitals. The antibiotic sensitivity rate was 100% for meropenem and imipenem in both ESBL and non-ESBL groups, followed by gentamicin with a sensitivity of 99.5% and 100% in the non-ESBL and ESBL groups, which showed the highest sensitivity against fluoroquinolones, cephalosporins, and other antibiotics [17]. Their results were consistent with ours, indicating the sensitivity of amikacin in both groups. A study titled “Escherichia coli and *Klebsiella pneumoniae* Sensitivity/Resistance Pattern towards Antimicrobial Agents in Primary and Simple Urinary Tract Infection Patients Visiting University Hospital of Jamia Hamdard New Delhi” by M. Rizwan (2018) examined 14 cases with UTIs. *E. coli*, followed by *Klebsiella pneumoniae*, were the commonest strains. *E. coli* showed the highest resistance against ampicillin, and then co-trimoxazole, norfloxacin, ciprofloxacin, gentamicin, tetracycline, and ceftazidime, respectively, whereas amikacin and nitrofurantoin caused the least resistance [17]. Our Study showed the therapeutic efficacy of a single dose of amikacin every 48 h. The main challenge of using aminoglycosides is their toxici effects. Their nephrotoxicity is reported 8–14%, which increases with higher dosages. They also have a treatment duration of ten days or more, or require the simultaneous administration of nephrotoxic compounds [1]. Our patients’ renal function was assessed by frequent serum creatinine assessments before, during, and following the treatment.

## Conclusion

The results of this study showed that treatment with amikacin, 1 g q48h, or meropenem, 1 g q8h, for one week, has the same results. Furthermore, treatment for UTIs with ESBL positive and ESBL negative cases should be the same, and amikacin can be used once every 48 h to treat UTIs. This treatment is less expensive and can be administered on an outpatient basis.

### Limitations

One of the limitations of this plan is the lack of taking more samples due to insufficient funds and also the examination of other samples except urine samples. Also, the examination of resistance genes in the samples could be examined, but it was not possible due to time and budget limitations.

## Data Availability

All data generated or analyzed during this study were included in this article but the raw data are available from the corresponding author on reasonable request.

## Abbreviations

UTIs: Urinary tract infections
*E. coli*: *Escherichia coli*
ESBL: Spectrum beta-lactamas
GFR: Glomerular filtration rate
EMB: Eosin methylene blue
SIM: Sulfide indol motility
TSI: Triple sugar iron agar
CLSI: Clinical and Laboratory Standards Institute
NCBL: Non carbapenem beta-lactams
APN: Acute pyelonephritis
